# *Apharinodesbaixiensis* sp. nov., a new species from Guangdong, China (Coleoptera, Staphylinidae, Pselaphinae)

**DOI:** 10.3897/BDJ.12.e139609

**Published:** 2024-11-18

**Authors:** Jiang Zhu, Cheng-Bin Wang

**Affiliations:** 1 State Environmental Protection Key Laboratory of Urban Ecological Simulation and Protection, South China Institute of Environmental Sciences, MEE, Guangzhou, China State Environmental Protection Key Laboratory of Urban Ecological Simulation and Protection, South China Institute of Environmental Sciences, MEE Guangzhou China; 2 Engineering Research Center for Forest and Grassland Disaster Prevention and Reduction, Mianyang Normal University, Mianyang, China Engineering Research Center for Forest and Grassland Disaster Prevention and Reduction, Mianyang Normal University Mianyang China

**Keywords:** Pselaphine beetle, Hybocephalini, new species, morphology, taxonomy, Indomalayan Realm

## Abstract

**Background:**

The genus *Apharinodes* Raffray, 1890 (Coleoptera, Staphylinidae, Pselaphinae, Hybocephalini) includes four known species occurring in East and Southeast Asia.

**New information:**

A new species, *Apharinodesbaixiensis* **sp. nov.**, is described from Heyuan City, Guangdong Province, China. Important morphological characters of the new species are illustrated by colour plates.

## Introduction

The genus *Apharinodes* Raffrary, 1890 belongs to the tribe Hybocephalini (Coleoptera, Staphylinidae, Pselaphinae), which was erected by Raffray (1890a) for a single species *Apharinodessquamosa* Raffrary, 1890 from Singapore. Later, Raffray (1890b) described the second species *Apharinodesmiranda* Raffrary, 1895, also occurring in Singapore. The third species, *Apharinodespapageno* Nomura, 1989 from Japan, was reported by Nomura (1989) and Yin and Jiang (2017) described the only species of the genus occurring in China, *Apharinodessinensis* Yin & Jiang, 2017.

*Apharinodes* is distinguished from the other genera of Hybocephalini by the combination of the following characters (Yin and Jiang 2017): 1) each antennal club is composed of only the enlarged antennomere 11, which is curved or impressed; 2) the entire body is covered by thickened squamous setae; 3) tergites IV and V are subequal in length.

Here, we describe and illustrate a new *Apharinodes* species from Guangdong Province, China, which is in accordance with all the above characters.

## Materials and methods

The single male specimen (holotype) of the new species was relaxed and softened in hot water for 24 hours, then transferred to distilled water to clean, observe and dissect. In order to examine the male genitalia, the abdomen was detached and treated with a 10% solution of potassium hydroxide (KOH) for 12 hours, then transferred to distilled water to flush the remaining KOH and stop any further maceration. After examination, the body parts were mounted on a glass slide with BASO sealing adhesive for further studies. All photographs were taken using a Sigma FP-L camera with Mitutoyo M Plan Apo HR Microscopic lens and two Amaran 200ds lights as light sources. The final deep focus images were created with Helicon Focus 7.7.0 stacking software. Adobe Photoshop CC 2024 was used for post-processing. Abdominal tergites and sternites in the text were numbered in the morphological sense.

The holotype is deposited in the Insect Collection of Shanghai Normal University, Shanghai, China (**SNUC**).

Measurement criteria in millimetres (mm) are as follows: **antennae length**: length between the base of scape and the apex of antennomere 11; **body length**: from the frontal apex to the tip of the abdomen.; **elytral length**: length between the basal border and the apex of elytra along suture; **elytral width**: widest part of both elytra combined; **head length**: length between the anterior margin of mandibular apices and the anterior margin of pronotum along the mid-line; **head width**: widest part of head (including eyes); **pronotal length**: length of the pronotum along the mid-line; **pronotal width**: widest part of pronotum.

## Taxon treatments

### Apharinodes baixiensis

Zhu & Wang sp. nov.

68414648-693B-5572-888A-CBE0A3685841

A36F51D9-2F1E-44BA-B939-AA38C042D1F4

#### Materials

- **Type status:** Holotype. **Occurrence:** recordedBy: Jiang Zhu, Ru-Tian Ye & Zhen-Yu Piao; individualCount: 1; sex: male; lifeStage: adult; **Location:** country: China; stateProvince: Guangdong; verbatimLocality: Heyuan City, Zijin County, Baixi Provincial Nature Reserve, Tangpa Mountain [河源市紫金县白溪省级自然保护区烫耙岭]; verbatimElevation: 274 m; decimalLatitude: 23.707126; decimalLongitude: 115.175949; geodeticDatum: WGS84; **Event:** verbatimEventDate: 28.III.2024; **Record Level:** institutionCode: SNUC

#### Description

**Holotype Male.** Body 1.77 mm in length, 0.76 mm in width, widest at second visible adbominal tergite. Length of body parts (mm): head (0.38), eye (0.13), antennae (0.73), pronotum (0.37), elytra (0.54); width: head (0.38), eye (0.07), pronotum (0.37), elytra (0.68).

**Figure 1.**
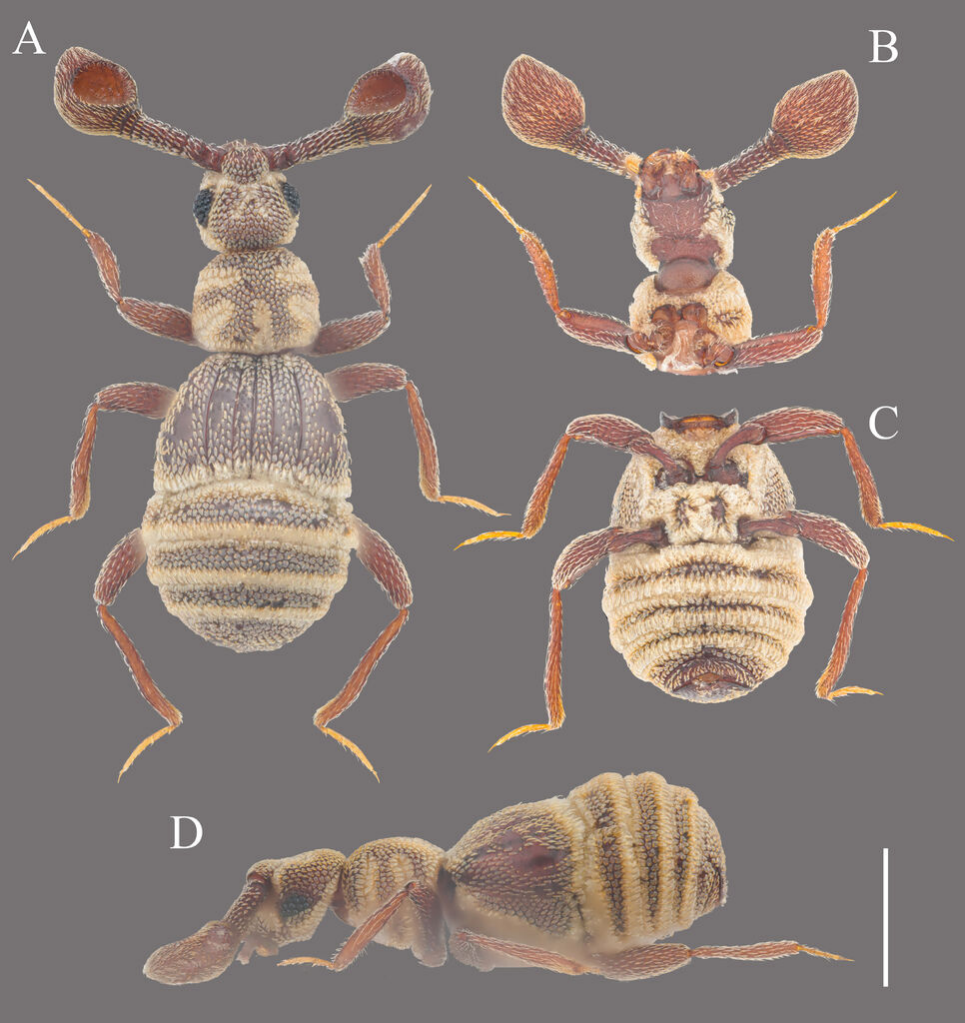
Habitus of *Apharinodesbaixiensis* **sp. nov. A** Dorsal view; **B, C** Ventral view; **D** Lateral view. Scale bar = 0.5 mm.

Habitus (Fig. 1A–D). Body dark reddish-brown, mostly covered by thick yellownish setae. Setae on legs and antennomere 11 finer; on other antennomeres, slightly stronger; on other body parts, enlarged to squamous scales.

Head subelliptical, as long as wide, widest across eyes. Dorsum entirly covered by yellownish scale-like setae; setae on clypeus slightly thinner. Clypeus slightly convex, nearly covering bases of antennae; eyes large, distinctly prominent, berry-shaped, each composed of about 20 facets; postocular margins shorter than eyes, roundly narrowed towards head base; frons with two connected semi-circular patches of squamous setae; maxillary palpi small and short, three-segmented, with distinct conical palpal cone; gular area flat.

Antennae robust, each with ten antennomeres which are close to each other, large club forming antennomere 11; antennomere 1 robust, cylindrical, about equal to length of antennomeres 2+3+4 combined; antennomeres 3 to 10 strongly transverse, successively wider, quite close to each other; antennomere 11 largest, about equal to length of antennomeres 2 to 10 combined, medio-dorsal surface with large, deep, circular excavation, nearly bowl-shaped, width/length = 0.83.

Prothorax. Pronotum width equal to length, subcylindrical, broadest at middle, nearly straight from middle to base, narrowed from middle towards apex. Surface fully covered by dense squamous scales, has two lateral pairs of squamous bands of setae and one median longitudinal band, nearly equal in length.

Elytra convex, wider than long, posterior margin with band of dense scale-like setae, two basal foveae covered by squamous setae, with weak discal and sutural striae. Metathoracic wings lacking. Mesoventrite densely covered with small squamous setae; metaventrite densely covered with setae at middle, areas posterior to mesocoxae with two longitudinal projections that extend to metaventral posterior margin.

Legs slightly lighter than other parts in colour, densely covered by hair-like setae. Inner claw of each tarsus highly degraded into seta shape.

**Figure 2.**
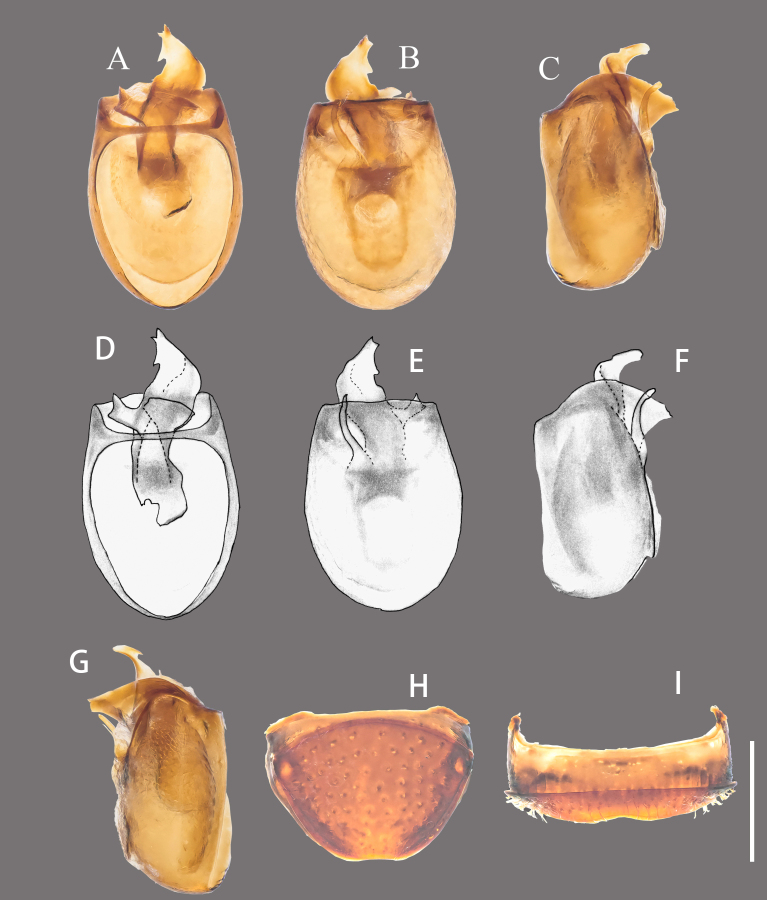
Details of male *Apharinodesbaixiensis* sp. nov. **A** Aedeagus, dorsal view; **B** Ditto, ventral view; **C** Ditto, left lateral view; **D** Ditto, dorsal view, line drawing; **E** Ditto, ventral view, line drawing; **F** Ditto, left lateral view, line drawing; **G** Ditto, right lateral view; **H** Tergite VIII; **I** Sternite VIII. Scale bar = 0.2 mm.

Abdomen subglobose, wider than long; tergite IV as long as V, with pair of lateral foveae, tergites VI and VII slightly shorter than V, tergite VIII (Fig. 2H) sparsely punctured, rather slightly emarginate at posterior margin; sternites IV to VII successively shorter, sternite VIII (Fig. 2I) shallowly emarginate at middle of posterior margin, sternite IX composed of paired membranous structures.

Aedeagus (Fig. 2A–G) 0.27 mm long, median lobe broad, with large basal capsule, broadly emarginate along apical margin; parameres invisible, probably lacking; endophallus composed of two sclerites in vertical arrangement, dorsal sclerite spina-like, ventral sclerite much stronger, spiral-shaped and slightly curved, more sclerotised laterally.

**Female.** Unknown.

#### Diagnosis

*Apharinodesbaixiensis* sp. nov. can be easily distinguished from any other congeners by the combination of the following characters: 1) antennomere 11 bowl-shaped impression in dorsal view (in *A.sinensis*, antennomere XI with deep, rounded excavation generally); 2) prothorax has two lateral pairs of squamous bands of setae and one median longitudinal band, nearly equal in length (in *A.sinensis*, anterolateral patches of squamous setae on pronotum shorter than basolateral patches); 3) Inner claw of each tarsus highly degraded into seta shape (in *A.sinensis*, inner claw also degraded into seta shape, but more developed); 4) frons with two separate semi-circular patches of squamous setae; 5) endophallus composed of two sclerites in vertical arrangement, the dorsal sclerite spina-like; the ventral sclerite much stronger, spiral-shaped and slightly curved, more sclerotised laterally, parameres invisible, probably lacking (in *A.sinensis*, endophallus composed of three long sclerites, parameres short and thick, each with three thick apical setae).

#### Etymology

The specific epithet is from the Chinese name (Pinyin) of the type locality "Baixi Provincial Nature Reserve". The name is an adjective.

#### Distribution

China (Guangdong).

## Supplementary Material

XML Treatment for Apharinodes baixiensis
